# Social anxiety prediction model for nursing students based on machine learning: a cross-sectional survey

**DOI:** 10.3389/fpsyt.2025.1721618

**Published:** 2025-12-12

**Authors:** Fang Wang, Pingping Xu, Yelin Huang, Li Liu, Liuliu Kong, Fan Yang

**Affiliations:** 1 Health Management College, Xianning Vocational and Technical College, Xianning, Hubei, China; 2 Endoscopy Center, Wuhan Children's Hospital, Tongji Medical College, Huazhong University of Science and Technology, Wuhan, Hubei, China; 3 Encephalopathy Department, Xian'an District Hospital of Traditional Chinese Medicine, Xianning, Hubei, China; 4 Nursing Department, Jinan Yinfei Hospital, Jinan, Shandong, China; 5 Operating Room, Nanjing Gaochun People's Hospital, Nanjing, Jiangsu, China

**Keywords:** nursing students, social anxiety, machine learning, prediction model, random forest

## Abstract

**Background:**

The purpose of this study is to use a variety of machine learning (ML) algorithms to build a risk prediction model for nursing students’ social anxiety, select the optimal model, and identify risk factors.

**Methods:**

The cross-sectional survey was conducted among nursing students at 10 universities from September to December 2024. A total of 2024 nursing students were included in this study. Nine acceptable features were selected through Logistic analysis. We developed and evaluated seven ML models: Logistic regression (LR), Elastic net (EN), k-nearest neighbors (KNN), Decision tree (DT), Extreme gradient boosting (XGBoost), Support vector machine (SVM), Random forest (RF).

**Results:**

The area under the Area Under Curve (AUC: 0.71) of the random forest model was the highest among the 7 models that predicted nursing students’ social anxiety. The most important characteristics that predicted social anxiety in nursing students included Sleep condition, alexithymia, depression, education level, and religious belief.

**Conclusion:**

Our findings suggest that ML models, specifically random forests, can best predict the risk of social anxiety among nursing students.

## Introduction

Social anxiety disorder is the third most common disorder in the general population (1).

Epidemiological studies have reported prevalence rates ranging between 7% and 13% in Western nations, while figures of approximately 10% and 11.7% have been documented in India and Saudi Arabia respectively (2). Social anxiety is defined as an irrational and excessive fear response elicited by interpersonal communication and social performance situations. This condition is characterized by intense affective manifestations (i.e., tension or distress) and is frequently accompanied by behavioral avoidance patterns in social contexts (3). Nursing is recognized as a profession in which significant emphasis is placed on social interaction and interpersonal relationships within clinical environments. Effective healthcare delivery in this field is dependent upon close communication and multidisciplinary collaboration being established among nurses, patients, caregivers, physicians, and allied health professionals (4). Nursing students often need to establish communication with teachers, doctors, patients, and their families during their internships, such as various operations, departmental assessment, health education for patients, and speaking in professional learning activities (5). Frequent exposure to these environments can easily lead to social anxiety. Rehab and Ahmad found in their research that the incidence of social anxiety among nursing students ranged from 28.15% to 47.5% (6, 7).

The presence of social anxiety in nursing students has been identified as a matter of particular concern due to its potential to impede the acquisition of essential clinical competencies, the establishment of therapeutic patient relationships, and effective participation in interdisciplinary healthcare teams. Furthermore, this condition has been associated with the development of multiple adverse psychological consequences, including diminished self-esteem, depressive symptoms, persistent fear responses, and feelings of social isolation (8, 9), as well as depression. The predictive validity of traditional scoring systems for social anxiety may be limited due to the multifactorial nature of its underlying mechanisms and the heterogeneity of contributing factors. This methodological constraint is attributed to the incorporation of an insufficient number of variables within conventional assessment frameworks. Machine learning (ML) algorithms are suitable for all types and sizes of data and have attracted great attention in developing patient-centered predictive/prognostic models (10). These models help optimize treatment plans and facilitate the monitoring and managing health conditions.

In our research, we chose the commonly used machine learning models: logistic regression (LR), elastic network (EN), K-nearest neighbor (KNN), decision tree (DT), extreme gradient enhancement (XGBoost), support vector Machine (SVM), and Random Forest (RF). The core of XGBoost is to adopt the integrated idea of boosting methods, generate weak learners by optimizing the structured loss function, and improve the performance of the algorithm through techniques such as pre-sorting and weighted quantiles to prevent overfitting. To improve the generalization ability of the model (11). SVM is used to construct a model for limited data, so that the model can achieve the effect of minimizing structural risk (12). During the process of model construction, the best compromise position is sought between the complexity of the function and the accuracy of data analysis, so as to obtain the best generalization ability (13). DT is a classic classification method, which is an algorithm for summarizing classification rules from the training dataset (14). This method has relatively low requirements for the data. As it divides the independent variables one by one, it has achieved remarkable results in fields such as classification, prediction, and rule extraction at present (14). EN is formed through training and learning from a large amount of source data and continuous iterative calculations. It can fit the nonlinear characteristics of the source data (15). Using this model, highly accurate predictions can be made for the characteristics of unknown data. KNN is a type of classification algorithm. It can supervise the machine to perform calculations and use Markov distance, Euclidean distance, etc., to minimize the similarity, thereby determining the classification of a certain data to be measured (16). Logistic regression is used to convert the result of a certain continuous value obtained by linear regression into a probability value with an interval, and then handle classification problems based on the obtained probability value (17). RF is an algorithm capable of fusing multiple decision trees and belongs to an ensemble algorithm (18). The operation is simple. Whether in the training process or parameter adjustment, it can be carried out quickly. The RF estimation error is combined to evaluate the fitting and prediction accuracy of the combined tree learner (19, 20). Grounded in the Health Ecological Model (21), which conceptualizes health outcomes as the product of multi-level and interacting factors, this study identified an initial set of feature variables for the ML models through a synthesis of this theoretical framework and a review of relevant literature (5, 22). We subsequently conducted a cross-sectional survey among nursing students to develop and test the applicability of ML models in predicting the risk of social anxiety. Furthermore, we analyzed and interpreted the importance of the input variables to identify the most significant predictors of social anxiety in this population.

## Methods

### Participants

The cross-sectional survey was conducted among nursing students at 10 universities in China from September to December 2024. Before distribution, the researchers explained the purpose of the study to all participants, informing them of the main content and confidentiality of the survey data. The nursing teachers distributed the electronic questionnaire; the informed consent statement was attached to the cover. Participants can only proceed with the survey if they agree, while those who refuse will be directed to an opt-out page. Informed consent was obtained from all participants. The study was done anonymously and voluntarily for 20 minutes. Before starting, this study was approved by the Biomedical Ethics Committee of China. We confirm that all procedures follow the relevant guidelines and regulations, including the Declaration of Helsinki. A total of 2,044 students completed the survey.

#### Inclusion criteria

(1) Nursing students; (2) Agree to participate in this study.

#### Exclusion criteria

(1) Physically disabled; (2) Those who ask for leave or are absent from classes during the investigation period;(3) Provided identical answers across all scale items; (4)Had missing data on key variables.

### Sample size

Sample size was calculated based on the principle that event per variable (EPV) ≥ 10 (23). 18 variables were expected to be included in this study, considering a loss to follow-up rate of 10%-20%, so the minimum sample size for modeling was 178. Since the modeled sample size represents 70% of the total sample, the total sample size is at least 283. After applying listwise deletion to the initial 2,044 completed surveys to remove responses with missing data or uniform answering patterns, 2,024 valid cases were retained, yielding an effective response rate of 99.02%.

### Ethical considerations

The Biomedical Ethics Committee approved the study of China and was conducted in accordance with the Declaration of Helsinki. All participants voluntarily agreed to participate and signed an informed consent form, and their personal information was anonymized. They were also informed of their right to refuse participation in the study at any stage.

### Measures

#### Demographic characteristics

The general information questionnaire was developed based on the Grounded in the Health Ecological Model (21) and a comprehensive literature review (5, 22). It included key demographic and behavioral variables such as gender, age, educational level, clinical internship experience, and drinking habits, among others.

#### Center for epidemiological studies depression scale

Depressive symptoms among nursing students were assessed using the Center for Epidemiological Studies Depression Scale (CES-D). Developed in 1977, the CES-D is a widely used 20-item self-report instrument designed to screen for depressive symptomatology in the general population (24). Each item is rated on a 4-point Likert scale from 0 (“rarely or none of the time less than 1 day”) to 3 (“most or all of the time 5–7 days”), resulting in a total score ranging from 0 to 60. The scale encompasses four dimensions: depressed mood, positive mood, somatic complaints, and interpersonal difficulties. According to the established cutoff criteria (24), a total score of ≤ 15 indicates no significant depressive symptoms, 16–19 suggests possible mild depression, and ≥ 20 reflects specific depressive symptoms. This scale has been well-validated in Chinese university student populations, demonstrating satisfactory reliability and validity (25, 26). In the present study, the scale exhibited excellent internal consistency, with a Cronbach’s α of 0.964.

#### Social interaction anxiety scale

Social anxiety was assessed using the 15-item self-report scale compiled by Leary (27). This unidimensional instrument measures the subjective tendency toward social anxiety, independent of observable behavior. Items are rated on a 5-point Likert scale ranging from 1 (“not at all”) to 5 (“very consistent”), yielding a total score between 15 and 75, with a commonly used cutoff score of 45 indicating the presence of social anxiety symptoms (28). This instrument has been extensively used in Chinese university student samples, with established reliability and validity. In the present study, it showed good internal consistency, with a Cronbach’s α of 0.828.

#### Toronto alexithymia scale-26

This study adopted the Toronto Alexithymia Scale revised by Chinese scholar Professor Yao et al (29). The scale contains 26 questions and four dimensions: the ability to describe emotions, recognize and distinguish between emotions and body feelings, fantasy, and extroverted thinking. A 5-point scale is used, from 1 (strongly disagree) to 5 (strongly agree). A score of 26 to 51 without alexithymia, 52 to 61 with alexithymia tendency, and 62 to 130 with alexithymia (30). This instrument has been extensively used in Chinese university student samples, with established reliability and validity (29). In this study, the Cronbach’s α for the sample was 0.938.

### Experimental environment

In this study, python 3.9.9 is used for all the experiments. The python dependency libraries used include: scikit-learn=1.3.2, pandas=2.1.4, numpy=1.26.3, XGBoost =2.0.3, matplotlib=3.9.4, shap=0.46.0.

### Statistical analysis

Statistical analyses were performed using SPSS 27.0. Categorical variables are presented as frequencies and percentages (n, %). Between-group comparisons were conducted using Chi-square tests, while potential risk factors were identified through logistic regression analysis (P<0.05). The processing dataset split into a training set (70%) and a testing set (30%). We then use these preselected features into seven different ML models: logistic regression (LR), elastic network (EN), K-nearest neighbor (KNN), decision tree (DT), extreme gradient enhancement (XGBoost), support vector Machine (SVM), Random Forest (RF). For each model, we select a set of hyperparameters that maximize the AUC of the ROC on the training set by using a 5-fold Cross Validation and Grid Search method, thus ensuring optimal performance and efficient prediction and comparison of the test set. The models have been cross- Validated in five times to ensure robustness and reliability. Comparing the models, we select the best model based on the AUC values and create the Shapley Additive exPlanations (SHAP) interpreter to calculate the SHAP values, representing each feature’s contribution to the predicted results, and we draw a SHAP summary diagram of RF model to illustrate the effects of model features.

## Results

### General characteristics of the participants

A total of 2024 nursing students were investigated in this study, including 406 males and 1618 females. Among them, 25.74% were rated as socially anxious. **Table 1** shows the baseline characteristics of the socially anxious group and the group without social anxiety. Two groups of patients were evaluated regarding education level, school type, Mother’s Educational Level, Single-person household, Sleep Condition, Religious Belief, Depression, and Alexithymia. The difference was statistically significant (P < 0.001). see **Table 1**.

**Table 1:** General characteristics of the participants.

Variables	Total	Social anxiety		χ²	P
		No	Yes		
Gender				3.393	0.065
Male	406	316	90		
Female	1618	1187	431		
Age(Years)				3.017	0.221
<20	1448	1061	387		
20-23	553	423	130		
≥24	23	19	4		
Ethnic Group				0.089	0.776
Han Chinese	2003	1488	15		
Ethnic Minority	21	15	6		
Educational Level				6.846	0.009**
Associate Degree or Below	1093	786	307		
Undergraduate Degree	931	717	214		
School type				8.940	0.003**
Public School	962	685	277		
Private School	1062	818	244		
Clinical Practicum				0.130	0.718
Yes	752	555	197		
No	1272	948	324		
Residence				0.591	0.442
Urban Area	876	658	218		
Rural Area	1148	845	303		
Household Monthly Income (∼)				5.79	0.055
≥5000	641	497	144		
2000-4000	861	632	229		
<2000	522	374	148		
Father’s Educational Level				3.238	0.198
Higher Education	307	235	72		
High School	455	348	107		
Junior High School or Below	1262	920	342		
Mother’s Educational Level				7.324	0.026*
Higher Education	262	198	64		
High School	336	268	68		
Junior High School or Below	1426	1037	389		
Single-person household				5.239	0.022*
Yes	466	365	101		
No	1558	1138	420		
Sleep Condition				68.776	<0.001***
Excellent	1222	977	245		
Fair	627	432	195		
Poor	175	94	81		
Smoking				0.208	0.648
Yes	75	54	21		
No	1949	1449	500		
Alcohol drinking				0.029	0.865
No	1822	1354	468		
Yes	202	149	53		
Religious Belief				4.318	0.038*
Yes	128	105	23		
No	1896	1398	498		
Depression				51.772	<0.001***
Yes	1445	1137	308		
No	579	366	213		
Alexithymia				49.778	<0.001***
Yes	362	322	40		
No	1662	1181	481		
Sexual Orientation				2.737	0.098
Heterosexual	1878	1403	475		
Sexual minority	146	100	46		

*P<0.05, **P<0.01, ***P<0.001 .

### Multivariate binary logistic regression analysis

Taking the occurrence of social anxiety among nursing students as the dependent variable (No=0, Yes=1), Educational Level (Associate Degree or Below =0, Undergraduate Degree=1), School type (Public School=0, Private School= 1), Mother’s Educational Level (Junior High School or Below =0, High school=1, Higher Education=2), Single-person household (No=0, Yes=1), Sleep Condition (Excellent=0, Fair=1, Poor=2) logistic regression analysis was conducted on Religious Belief (Yes=0, No=1), Depression (No=0, Yes=1), and Alexithymia (No=0, Yes=1). Multivariate binary logistic regression analysis found that the Undergraduate Degree (OR = 0.756, P = 0.009, 95%CI: 0.613 to 0.932) was a protective factor for social anxiety in nursing students. Poor Sleep Condition (OR = 1.637, P < 0.001, 95%CI: 1.399 to 0.916), Depression (OR = 2.093, P < 0.001, 95%CI: 1.690 to 2.592), Alexithymia (OR = 3.093, P < 0.001, 95%CI: 2.184 to 4.381) was a risk factor for social anxiety in nursing students, as shown in **Table 2**.

**Table 2:** Multivariate binary logistic regression analysis of social anxiety in nursing students.

Risk factor	Reference factor	B	SE	Waldx^2^	P	OR	95%CI
Educational Level	Associate degree or below						
Undergraduate Degree		-0.280	0.107	6.832	0.009	0.756	0.613 to 0.932
School type	Public school						
Private School		-0.125	0.160	0.606	0.436	0.883	0.644 to 1.209
Mother’s Educational Level	Junior high school or below						
Higher Education		0.055	0.079	0.472	0.492	1.056	0.904 to 1.234
Single-person household	No						
Yes		0.172	0.137	1.587	0.208	1.188	0.909 to 1.553
Sleep Condition	Excellent						
Poor		0.493	0.080	37.686	<0.001	1.637	1.399 to 1.916
Religious Belief	Yes						
No		0.486	0.236	4.243	0.039	1.626	1.024 to 2.583
Depression	No						
Yes		0.739	0.109	45.843	<0.001	2.093	1.690 to 2.592
Alexithymia	No						
Yes		1.129	0.179	40.410	<0.001	3.093	2.184 to 4.381

### Performance evaluation

**Figure 1:**
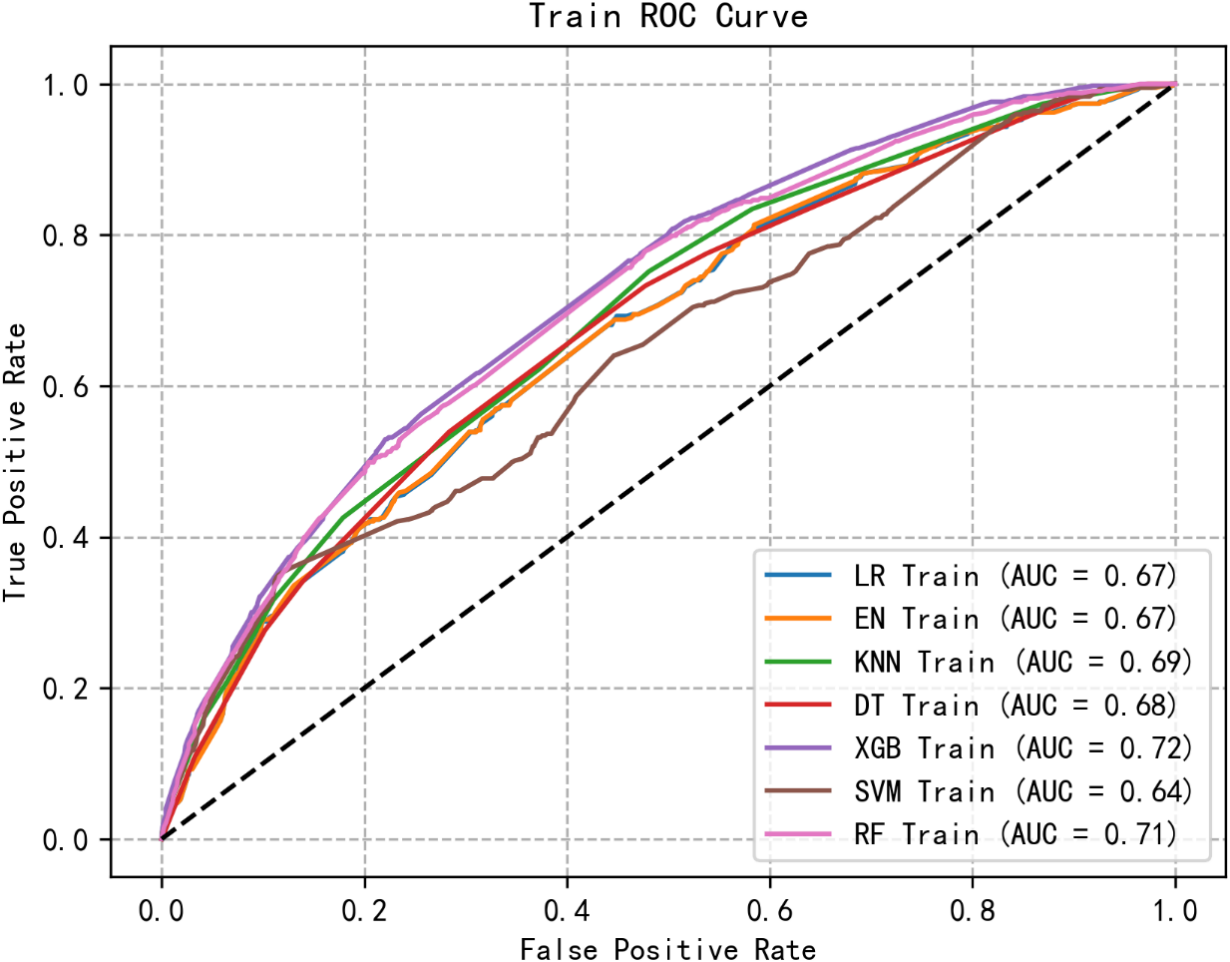
The receiver operating characteristic curves of 7 machine learning models in training set. LR, logistic regression; XGBoost, extreme gradient boosting; DT, decision tree; SVM, support vector machine; KNN, k-nearest neighbors; RF, random forest; EN, elastic net.

**Figure 2:**
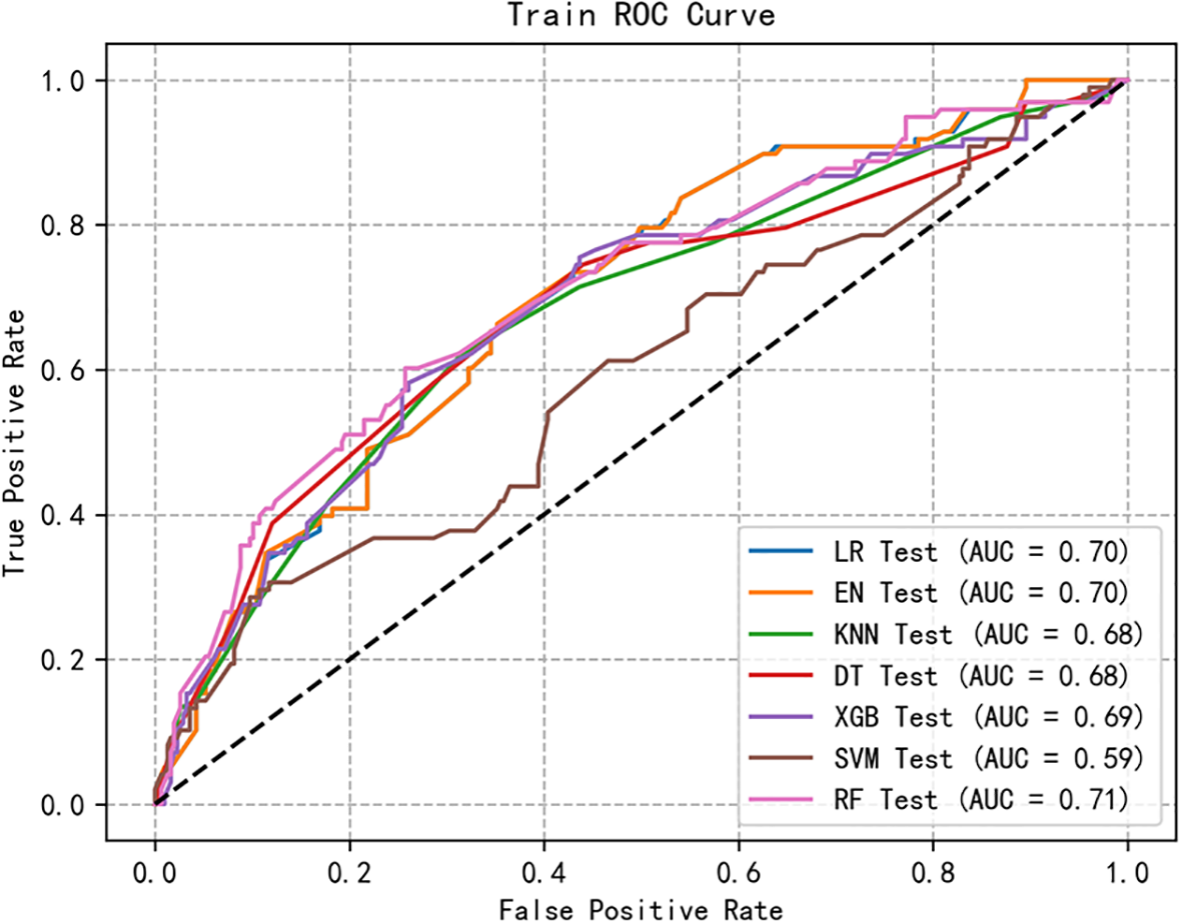
The receiver operating characteristic curves of 7 machine learning models in testing set. LR, logistic regression; XGBoost, extreme gradient boosting; DT, decision tree; SVM, support vector machine; KNN, k-nearest neighbors; RF, random forest; EN, elastic net.

**Figure 3:**
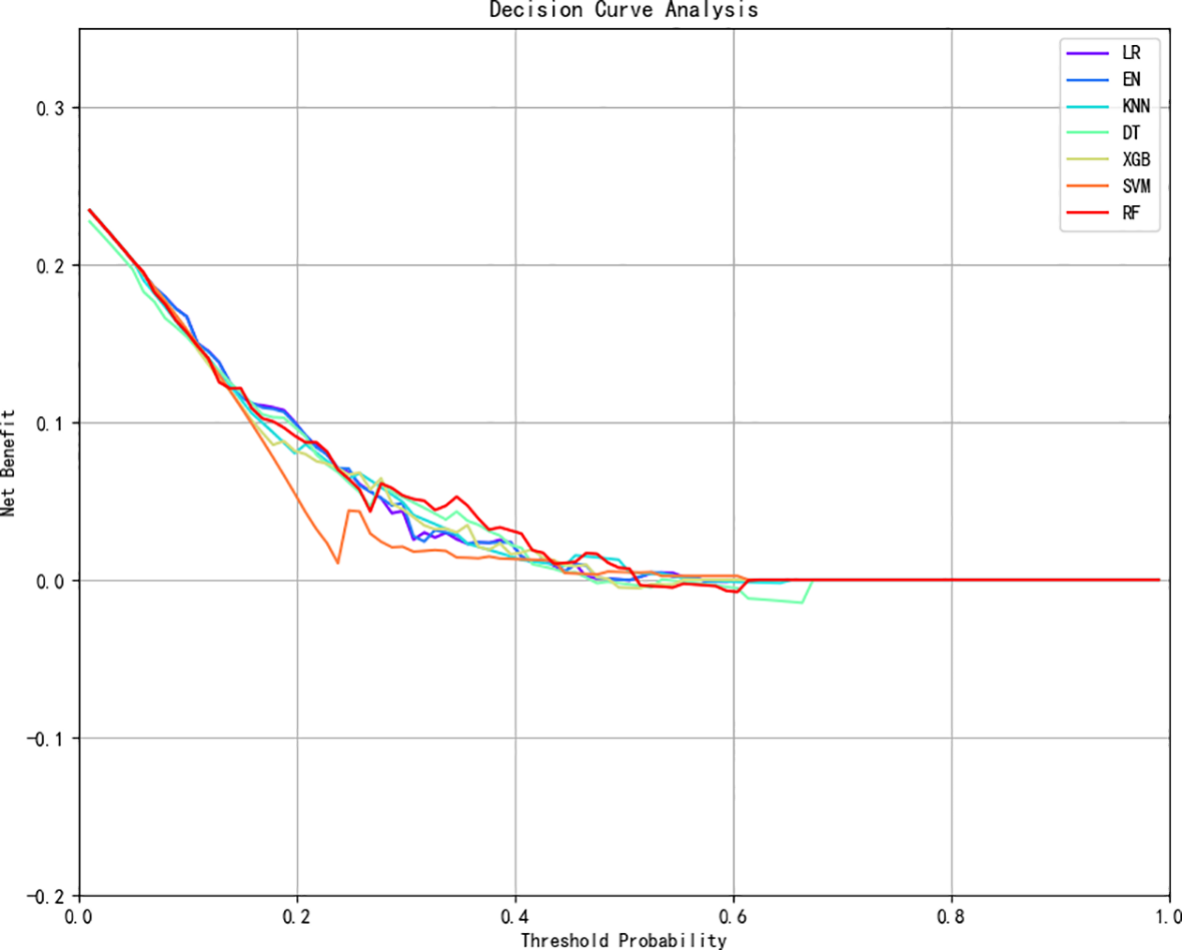
DCA curves for seven machine learning models. LR, logistic regression; XGBoost, extreme gradient boosting; DT, decision tree; SVM, support vector machine; KNN, k-nearest neighbors; RF, random forest; EN, elastic net.

**Figure 4:**
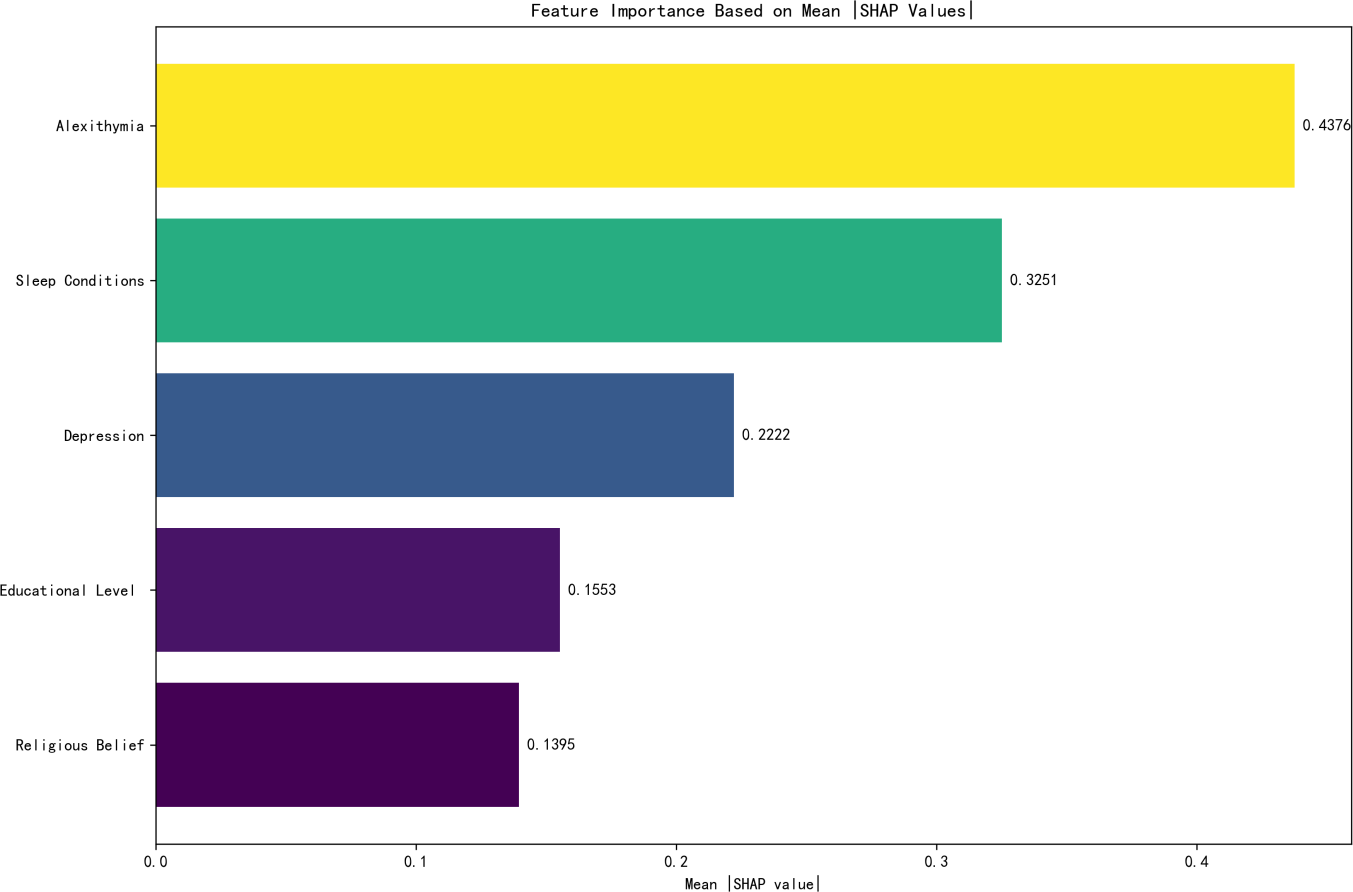
Feature importance ranking.

**Figure 5:**
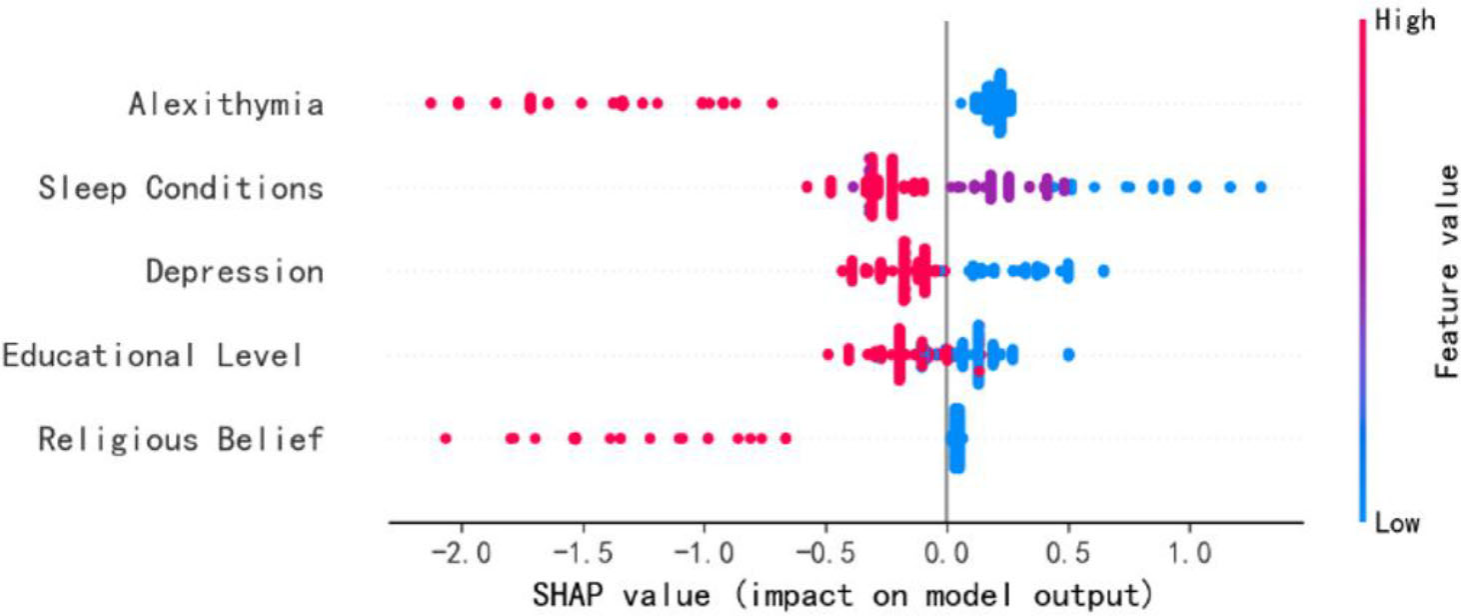
SHAP value (impact on model output).

We retained five significant and highly correlated individual characteristics through Logistic correlation analysis. Using these five variables, we developed seven ML models to predict the risk of social anxiety in nursing students. **Figures 1**, **2** shows the ROC of all model test sets and validation sets, whose prediction and discrimination are represented by AUC. The test set RF model has the highest AUC (0.71), followed by LR (0.70), EN (0.70), XGBoost (0.69), KNN (0.68), DT (0.68), and SVM (0.59). DCA was used to evaluate the clinical applicability of all models further. DCA was used to assess the clinical efficacy of the prediction model. In most threshold ranges, RF partially or completely overlaps with other models, indicating no significant difference in net benefits, however, at about 0.2-0.4. Within the threshold range, the net benefit of the RF model is significantly higher than that of the other models (**Figure 3**). In addition, we calculated the accuracy, sensitivity, specificity and confusion matrices of all models (**Table 3** and **Appendix 1: Figures 1-7**). The RF model had the highest accuracy at 0.763. Considering other indicators, the overall RF performance is better.

**Table 3:** Comparison of prediction models.

Prediction model	Accuracy	Precision	F1	Sensitivity	Specificity	Recall	Balanced accuracy	95%CI
Logistic regression (LR) training group	0.742	0.542	0.154	0.090	0.973	0.090	0.622	0.603,0.684
Logistic regression (LR) test group	0.758	0.500	0.093	0.051	0.984	0.051		
Elastic net (EN) training group	0.742	0.543	0.154	0.090	0.973	0.090	0.615	0.596,0.684
Elastic net (EN) test group	0.758	0.500	0.093	0.051	0.984	0.051		
k-nearest neighbors (KNN) training group	0.746	0.597	0.163	0.095	0.977	0.095	0.583	0.592,0.668
k-nearest neighbors (KNN) test group	0.763	0.600	0.111	0.061	0.987	0.061		
Decision tree (DT) training group	0.743	0.541	0.181	0.109	0.967	0.109	0.574	0.660,0.689
Decision tree (DT) test group	0.765	0.600	0.159	0.092	0.981	0.092		
Extreme gradient boosting (XGBoost) training group	0.746	0.762	0.072	0.038	0.996	0.038	0.618	0.633,0.678
Extreme gradient boosting (XGBoost) test group	0.753	0.250	0.020	0.010	0.990	0.010		
Support vector machine (SVM) training group	0.748	0.603	0.167	0.097	0.977	0.097	0.574	0.647,0.694
Support vector machine (SVM) test group	0.768	0.667	0.146	0.082	0.987	0.082		
Random forest (RF) training group	0.749	0.615	0.192	0.114	0.975	0.114	0.616	0.627,0.682
Random forest (RF) test group	0.763	0.615	0.192	0.114	0.975	0.114		

Balanced Accuracy is mean recall across categories.

### Feature importance ranking

To visually present the selected variables, we employ the best feature ranking to show the importance of each factor (**Figure 4**). Alexithymia significantly influenced model output, followed by sleep condition, depression, educational level, and religious belief. **Figure 5** provides a more detailed view of the impact of each feature on individual predictions. Alexithymia exhibited a significant positive dose-response relationship, with SHAP values continuously increasing as scores rose. The negative impact on the model output is the greatest during periods of poor sleep quality. As the quality improves, this impact tends to stabilize. However, the effect sizes of depression, educational level, and religious belief are relatively small.

## Discussion

Our results found that the incidence of social anxiety among nursing students was 25.79%, which was lower than Wang’s (31) survey result (34.67%). However, it is still higher than Nader’s (32) meta-analysis of adolescents (17%). It shows that the social anxiety of nursing students is still at a high level. The nursing profession attaches more and more importance to the education and training of high social intelligence. Still, professional characteristics such as the imbalance of male and female ratio, traditional social concepts, low professional identity, and little emotional support lead to social anxiety. Secondly, when nursing students enter the clinical practice stage, they face a huge psychological impact, such as role identity change, and are easily exposed to different occasions and contact with other patients, so they become a group with a high incidence of social anxiety. Therefore, nursing educators and managers should conduct social anxiety assessments for nursing students in a timely manner and hold regular psychological lectures to reduce the occurrence of social anxiety among nursing students and promote their physical and mental health. In addition, in our study, we selected five acceptable traits and built seven ML models to predict the risk of social anxiety in nursing students. The RF model has the highest AUC and good clinical applicability among the seven models. The most important characteristics that predicted social anxiety in nursing students were sleep status, alexithymia, depression, education level, and religious affiliation.

Numerous studies have established that sleep disturbances—such as insomnia, nightmares, sleepwalking, and excessive daytime sleepiness—contribute to the onset, recurrence, and persistence of mental health issues (33, 34). While prior research has identified sleep condition as a factor influencing social anxiety among nursing students (35), our study further reveals that sleep status emerged as the second most important predictor of social anxiety in this population. This finding is supported by neurocognitive explanations suggesting that sleep deprivation alters neural network activity—for instance, heightening sensitivity in brain regions associated with signaling social rejection—which may subsequently promote social withdrawal and avoidance behaviors (36). Therefore, improving sleep quality should be regarded as a crucial component in strategies aimed at alleviating anxiety symptoms among nursing students.

Our findings identified alexithymia as the primary predictor of social anxiety among nursing students. Alexithymia is characterized by deficits in emotion awareness, including difficulties in identifying feelings, distinguishing between emotions and bodily sensations, reduced imaginative capacity, and an externally oriented cognitive style (37). According to the socio-formation mechanism, alexithymia originates from early childhood experiences and is reinforced through sociocultural and relational contexts (38). As a manifestation of impaired emotional cognition and regulation (39), alexithymia is considered a risk factor for various psychological issues and maladaptive behaviors (40–42). In social situations, individuals with high levels of alexithymia often appear indifferent or detached due to their limited ability to recognize and express emotions, which impedes the formation of healthy interpersonal relationships and exacerbates social anxiety (43). Moreover, their limited imaginative and introspective capacities lead to the use of maladaptive coping strategies—such as suppression—when facing stressful events or internal conflicts (44), further increasing anxiety and impairing empathy (45, 46). In light of these results, we recommend that educational institutions implement mental health initiatives—such as psychoeducational lectures, group counseling, and psychological workshops—to disseminate knowledge on emotional health and guide students in improving emotional expression, thereby potentially reducing the prevalence and impact of alexithymia.

Our findings are consistent with previous reports by Hou (47) and Ye (48), reinforcing depression as a significant predictor of social anxiety among nursing students. In our analysis, it was ranked as the third most influential feature. Clinically defined by persistent sadness, hopelessness, and anhedonia (49), depression is theorized to drive dysfunctional social behaviors through excessive negative interpretation biases (50). The stress resulting from these impaired interactions is hypothesized to further consolidate such biased cognitive patterns, potentially creating a self-sustaining cycle of cognitive and social impairment (50). In addition, our study found that educational level is a factor affecting the social anxiety of nursing students, and the incidence of social anxiety among professional nursing students is significantly higher than that of undergraduate nursing students. On the one hand, postgraduates’ sense of social efficacy gradually increases with age, thus promoting the development of social ability. On the other hand, graduate students are more involved in social activities and work and are more exposed to social situations. Exposure itself will bring about the desensitization effect of social anxiety (51). Secondly, the junior college system is shorter than the undergraduate system. Theoretically, junior college nursing students spend less time in school than undergraduate nursing students, and their theoretical knowledge and skill levels are often slightly inferior. Moreover, junior college nursing students are younger, so they are prone to lack confidence in clinical practice, leading to social anxiety in nursing students. Our results show that whether nursing students have religious beliefs or not is a factor affecting the social anxiety of nursing students. Religious belief is a kind of “kernel” promoting factor. On the level of social relations, social support is a kind of “external” support factor, and the two work together in people’s spiritual world (52). At the same time, the concept of “being kind to others and living in harmony” in religious teachings will enable individuals to achieve harmonious coexistence between people in daily life and thus obtain more social support, thereby reducing the social anxiety of nursing students (52). In addition, the influence of faith on subjective well-being cannot be ignored. Religious belief has a guiding function on individual thoughts and behaviors, and the spiritual protection function of religious culture is conducive to making human beings perceive happiness, thus reducing the psychological pressure on nursing students in interpersonal communication (53). Therefore, school teachers or clinical teachers should pay more attention to the psychological dynamics of nursing students in practice, give psychological assessments to nursing students in time, pay attention to the characteristics of specialized nursing students, encourage the improvement of their academic qualifications, teach nursing students with different educational levels according to their aptitude, pay attention to the cultivation of their interpersonal skills, improve their ability to deal with things and their ability to withstand pressure and reduce their social anxiety.

## Strengths & limitations

To our knowledge, this study is the first to construct and compare the performance of seven different social anxiety risk prediction models for nursing students. In addition, our survey was a multicenter large-sample survey of 2,024 nursing students and combined machine learning techniques with demographic characteristics to predict nursing students’ social anxiety. However, some limitations to our study should not be ignored. First, the data sets used in this study are mostly self-dependent self-reports of nursing students, and the obtained data has certain errors. Second, because this study is cross-sectional, it cannot show causality, which is different from longitudinal research. Our model ignores social and behavioral factors, such as social support and lifestyle, critical to understanding social anxiety outcomes among nursing students. Incorporating these factors in future studies could enhance the power of the model. Finally, the dataset used in this study may have a situation of class imbalance. Although we have tried various methods to alleviate this problem (such as oversampling, undersampling, SMOTE, etc.), it still limits the effective prediction of the model. Future research can attempt other models, reinforcement learning, to further clarify the results of this study. The dataset used for modeling suffered from class imbalance, meaning there was a significant disparity in the ratio between high-risk and low-risk individuals for social anxiety. This may have influenced the estimation of certain performance metrics, such as the AUC. Although we comprehensively evaluated the models using multiple metrics (including Precision, Recall, and F1-Score) to mitigate this concern, future studies should seek to validate our models in larger and more balanced samples.

## Conclusion

The RF model has good clinical applicability and is expected to become an effective auxiliary tool for psychological screening of nursing students. Among the included variables, sleep state and alexithymia are the two most significant factors affecting the output of the RF model. However, the validity of this model in the external cohort and its potential to reduce social anxiety among nursing students remains to be determined.

## Data Availability

The original contributions presented in the study are included in the article/**Supplementary Material**. Further inquiries can be directed to the corresponding author.
