# Supplementary figures and images for: Social anxiety prediction model for nursing students based on machine learning: a cross-sectional survey
Source: Front Psychiatry. 2025 Dec 12;16:1721618. doi: 10.3389/fpsyt.2025.1721618 (PMC12766779; doi:10.3389/fpsyt.2025.1721618)

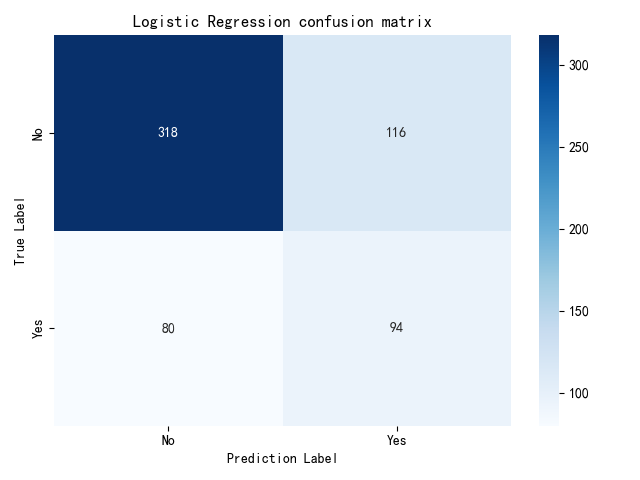

Supplement: Supplementary file 1 [file DataSheet1.zip › Appendix 1/Figure_1.png]

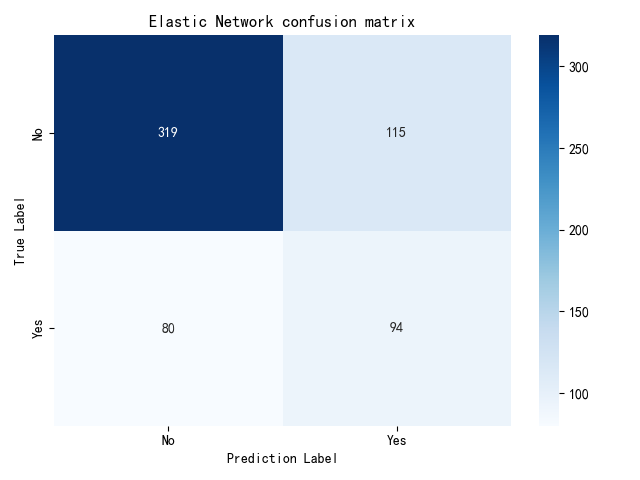

Supplement: Supplementary file 1 [file DataSheet1.zip › Appendix 1/Figure_2.png]

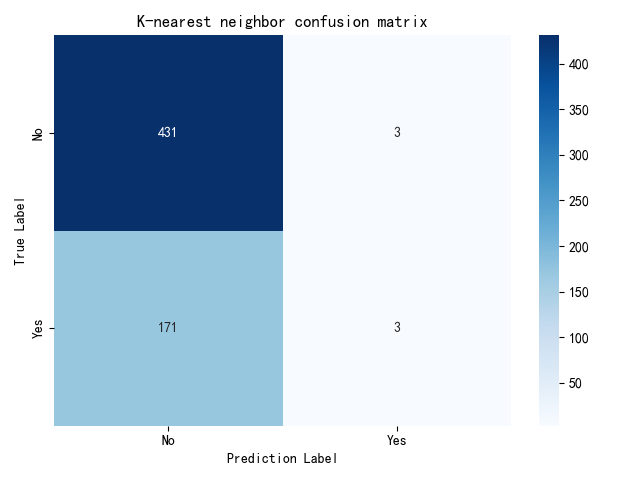

Supplement: Supplementary file 1 [file DataSheet1.zip › Appendix 1/Figure_3.png]

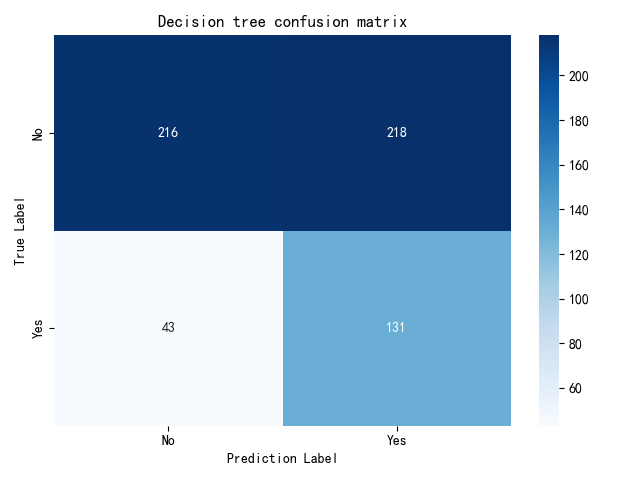

Supplement: Supplementary file 1 [file DataSheet1.zip › Appendix 1/Figure_4.png]

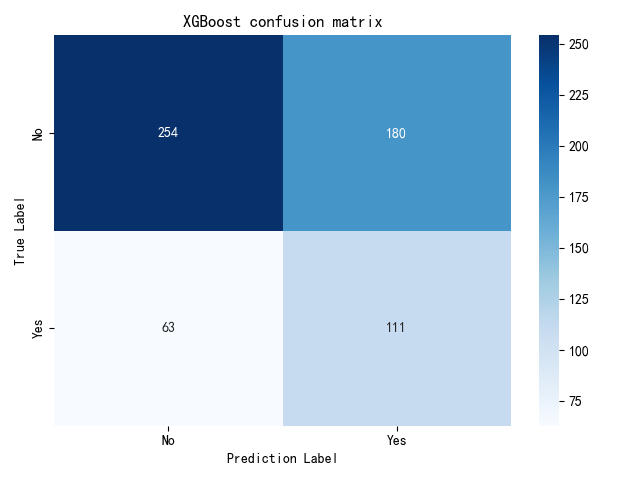

Supplement: Supplementary file 1 [file DataSheet1.zip › Appendix 1/Figure_5.png]

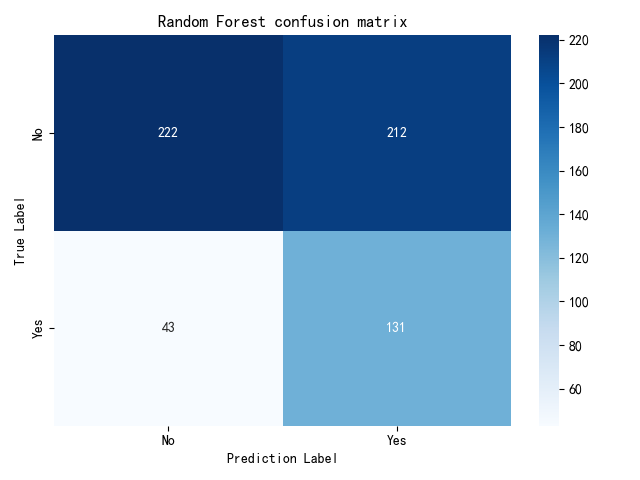

Supplement: Supplementary file 1 [file DataSheet1.zip › Appendix 1/Figure_6.png]

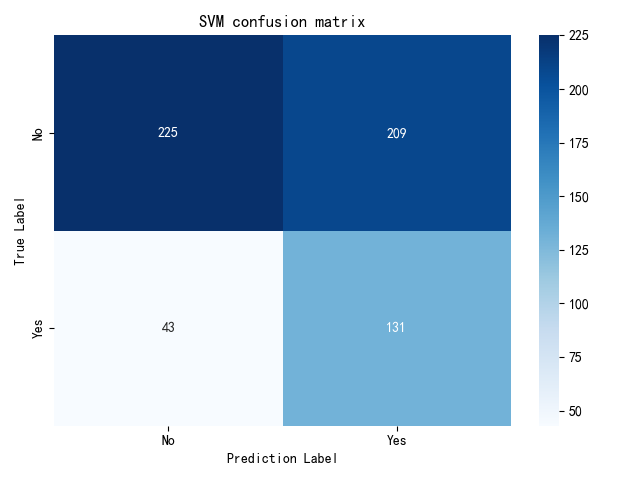

Supplement: Supplementary file 1 [file DataSheet1.zip › Appendix 1/Figure_7.png]
